# Dancing Megasperm

**DOI:** 10.5334/jbsr.1687

**Published:** 2019-01-09

**Authors:** Benjamin Leenknegt, Lucy Diss, Paul S. Sidhu

**Affiliations:** 1 University Hospital, Ghent, BE; 2 King’s College Hospital, London, GB

**Keywords:** Ultrasound, Spermatic duct obstruction, Filarial dance, Dancing megasperm

A 55-year old man is referred to the ultrasound department by his general practitioner for a follow-up examination of previously diagnosed testicular microlithiasis. A history of bilateral vasectomy was indicated by the patient.

On ultrasound, the presence of testicular microlithiasis was confirmed. Additionally, a diffuse bilateral enlargement of the epididymis with multiple small cystic dilatations was demonstrated (Figure 1). Within these dilatations, irregular small hyperechoic particles were depicted (Figure 2). These particles demonstrated a continuous oscillating to-and-fro movement (Figure 3 – movie clip). The finding is in keeping with “dancing megasperm”.

**Figure 1:**
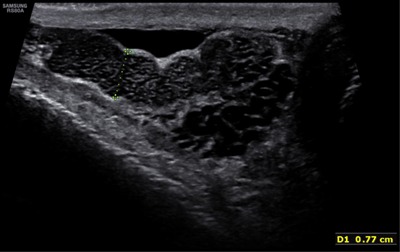
Enlargement of the right epididymis with multiple small cystic dilatations.

**Figure 2:**
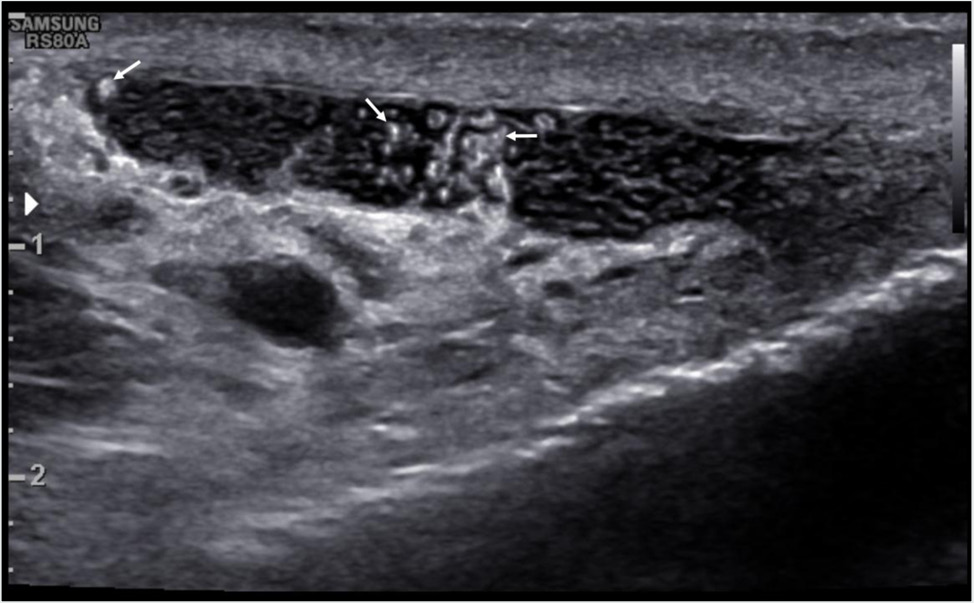
Irregularly shaped, hyperechoic particles (arrows) within the cystic dilatations of the epididymis.

**Figure 3:**
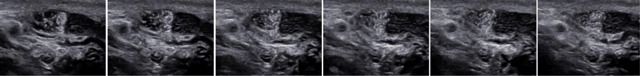
Selected images of an ultrasound film. Oscillating movement of the hyperehoic particles.

“Dancing megasperm” is believed to represent clusters of agglutinated spermatozoa within dilated epididymal tubules in patients with an obstruction of the spermatic duct. These findings were first described as “filarial dance” in men affected with lymphatic filariasis, a parasitic disease, most commonly from the *Wuchereria bancrofti filarial roundworm. W. bancrofti* is endemic in tropical and subtropical regions. It is transmitted through mosquitoes infected with microfilaria, the larval form of the filarial worm. Following a mosquito bite, the microfilaria invade human lymphatic channels and develop into adult worms, producing and releasing microfilaria, which eventually invade the bloodstream. In the acute phase, filariasis presents as acute lymphangitis. The chronic presentation is described as “elephantiasis”, with lymph oedema of an affected limb resulting from adult worms obstructing the lymphatic channels, causing extreme enlargement of the affected limb. Filariasis may also affect the lymphatic ducts of the inguinal channel and the scrotal sac, causing epididymitis, hydrocele or scrotal swelling.

The sonographic finding of “filarial dance” was hypothesised to represent adult filarial worms or microfilaria within the epididymis. However, the encounter of very similar sonographic findings in nonendemic areas for filariasis made the hypothesis unlikely. This was endorsed by a number of observations. First, filarial worms are too large and microfilaria too small to represent the echogenic particles seen on ultrasound. Second, “filarial dance” was often found in men with negative blood findings for filariasis.

The majority of men with “filarial dance” in nonendemic areas had a surgical or medical history related to the epididymis. In most cases, there was a history of vasectomy or, less frequently, bacterial epididymitis. The unifying feature of men with “filarial dance” in endemic and nonendemic areas appeared to be the presence of a process causing spermatic duct obstruction. A post-mortem study on the epididymides in men with spermatic duct obstruction demonstrated dilated epididymal tubules containing large clumps of agglutinated spermatozoa. This observation supports the hypothesis that “filarial dance” most likely represents “dancing megasperm”.

In conclusion, “dancing megasperm” is defined as the sonographic finding of moving, hyperechoic particles within cystic dilatations of an enlarged epididymis in a patient with spermatic duct obstruction. The particles most likely represent clusters of agglutinated spermatozoa. Oscillating movement of the particles is attributed to the turbulence within an acoustic medium on ultrasound. The finding is of clinical relevance as moving, hyperechoic particles within cystic dilatations of an enlarged epididymis could be misinterpreted as epididymitis with micro-abscesses [1].

## Additional File

The additional file for this article can be found as follows:

10.5334/jbsr.1687.s1 Movie clip Oscillating movement of the hyperehoic particles within the cystic dilatations of the epididymis. https://youtu.be/iNECJLjcmbk.
